# Fasting substrate oxidation in relation to habitual dietary fat intake and insulin resistance in non-diabetic women: a case for metabolic flexibility?

**DOI:** 10.1186/1743-7075-10-8

**Published:** 2013-01-14

**Authors:** Madelaine T Carstens, Julia H Goedecke, Lara Dugas, Juliet Evans, Jacolene Kroff, Naomi S Levitt, Estelle V Lambert

**Affiliations:** 1 MRC/UCT Research Unit for Exercise Science and Sports Medicine, Department of Human Biology, Faculty of Health Sciences, University of Cape Town, PO BOX 115, , Newlands, Cape Town, 7725, South Africa; 2 Department of Preventive Medicine and Epidemiology, Strict School of Medicine, Maywood, IL, USA; 3 Division of Endocrinology & Diabetic Medicine, Department of Medicine, Faculty of Health Sciences, University of Cape Town, Cape Town, South Africa; 4 South African Medical Research Council, Parow, Cape Town, South Africa

**Keywords:** HOMA-IR, Insulin-sensitivity, Dietary fat intake

## Abstract

**Background:**

Metabolic flexibility described as “the capacity of the body to match fuel oxidation to fuel availability” has been implicated in insulin resistance. We examined fasting substrate oxidation in relation to dietary macronutrient intake, and markers of insulin resistance in otherwise healthy women, with and without a family history of diabetes mellitus (FH DM).

**Methods:**

We measured body composition (dual x-ray absorptiometry), visceral and subcutaneous adipose tissue area (VAT, SAT, using Computerised Tomography), fasting [glucose], [insulin], [free fatty acids], [blood lipids], insulin resistance (HOMA-IR), resting energy expenditure (REE), respiratory exchange ratio(RER) and self-reported physical activity in a convenience sample of 180 women (18-45 yrs). A food frequency questionnaire was used to assess energy intake (EI) and calculate the RER: Food Quotient (FQ) ratio. Only those with EI:REE (1.05 -2.28) were included (N=140). Insulin resistance was defined HOMA-IR (>1.95).

**Results:**

The Insulin Resistant (IR) group had higher energy, carbohydrate and protein intakes (p < 0.05) and lower PA levels than Insulin Sensitive (IS) group (P < 0.001), but there were no differences in RER or RER:FQ between groups. However, nearly 50% of the variance in HOMA-IR was explained by age, body fat %, VAT, RER:FQ and FH DM (adjusted R^2^ = 0.50, p < 0.0001). Insulin-resistant women, and those with FH DM had a higher RER:FQ than their counterparts (p < 0.01), independent of body fat % or distribution.

**Conclusion:**

In these apparently healthy, weight-stable women, insulin resistance and FH DM were associated with lower fat oxidation in relation to dietary fat intake, suggesting lower metabolic flexibility.

## Background

The World Health Organization estimates that nearly 60% of adult cases of diabetes, 1 in 5 cases of ischemic heart diseases, and between 8-42% of certain cancers may be attributable to adult obesity [1]. Obesity and diabetes have become major public health problems in both developed and developing countries [2-4]. This trend in obesity may be linked to changes in population levels of physical activity [5], along with a changing food environment, in which low cost, high energy density, high-fat foods are more freely available [6-8].

Consumption of energy dense/high fat diets is strongly and positively associated with overweight and insulin resistance, particularly when the excess body fat is located in abdominal region [9,10]. Nevertheless, the link between fat intake and overweight is not limited to the high-energy content of fatty foods, but has also been shown to be associated with a reduced capacity to oxidize dietary fat [11-14].

Metabolic flexibility has been defined as “a clear capacity to utilize lipid and carbohydrate fuels and to transition between them” in response to changes in dietary energy intake or circulating substrate concentrations [15]. A failure in metabolic flexibility may lead to not only the obese state, but to the metabolic sequelae of insulin resistance [16]. Metabolic flexibility as an ‘intrinsic property of skeletal muscle’ has been further supported by *in vitro* studies in which the capacity of glucose to suppress fat oxidation in cultured myotubes from muscle biopsy samples was inversely correlated with in vivo insulin sensitivity and positively correlated to % body fat and serum free fatty acid levels [17].

Metabolic flexibility in vivo is typically measured by quantifying the oxidative response to some metabolic challenge, such as insulin-stimulation [18] or short-term, high-fat feeding. For example, in a study [19], young, otherwise healthy men, with a family history of diabetes, had a lower fat oxidation in response to a 3-day, high-fat diet, when compared to the fat oxidation those without a family history of diabetes.

Evidence from epidemiological [20,21] and intervention studies, implicates total dietary fat, and in particular, dietary saturated fat in insulin resistance. The potential mechanisms underpinning the effect of dietary fatty acids on insulin resistance may include altered substrate oxidation and metabolic flexibility [22], modifications in the composition of cell membranes, and stimulation of pro-inflammatory pathways in the body, promoting chronic inflammation, and may be associated with increased risk for cancer, heart disease, stroke, diabetes, arthritis and auto-immunity [10].

In this study, we propose a pragmatic measure of metabolic flexibility by comparing fasting substrate oxidation, with habitual dietary macronutrient intake, for the purposes of screening. This measure may provide some potential targets for intervention, for the purposes of preventing Type 2 diabetes mellitus [23].

Therefore, we firstly hypothesized that the measure of fasting fat oxidation in relation to habitual dietary fat and fatty acid intake, as a proxy for metabolic flexibility, would be associated with obesity and insulin resistance in a group of apparently healthy women. We further hypothesized, that this relationship may be modulated by a family history of diabetes. Thus, the findings of the present study support the notion that interventions to improve metabolic flexibility, and thereby insulin sensitivity, should ideally target physical activity levels, and/or dietary fat content or quality.

## Methods

### Subjects

A convenience sample of 180 healthy premenopausal urban-dwelling white South African women between the ages of 18 to 45 years was recruited through the local media, as part of a larger study [24]. The protocol was approved by the Research Ethics Committee of the Faculty of Health Sciences, University of Cape Town. The study was explained to each participant prior to the start of the trial and informed, written consent was obtained from all subjects.

### Body composition

Body weight was measured, using a combination scale and stadiometer (Universal weight enterprise Detecto model BW-150, Taipei, Taiwan). Waist girth (at the level of the umbilicus) and hip girth (over the area of largest circumference) were measured in participants with a steel anthropometric tape (Rosscraft Innovations Incorporated, Canada) wearing light clothing and were also used to calculate the waist-to-hip ratio (WHR). Whole body composition was measured using dual-energy X-ray absorptiometer (DXA) (Hologic QDR 4500 Discovery-W with software version 4.40, Hologic Inc., Bedford, MA, USA) according to standard procedures. The co-efficient of variation (%) for fat-free mass, using this technique, is as low as 0.7%, and 1.67% for fat mass. Body fat distribution was measured using computerized tomography (CT) scans at the level of the L4-L5 lumbar vertebrae. Visceral and subcutaneous tissue areas (VAT and SAT, respectively) were quantified as described previously [25]. Due to logistical constraints DXA scans were only completed on 66 insulin sensitive and 70 insulin resistant subjects and CT scans on 55 insulin sensitive and 58 insulin resistant subjects.

### Blood pressure, glucose tolerance and lipoprotein profiles

Blood pressure (BP, mmHg) was taken as the average of three readings from the left arm, after subjects rested quietly for 5 min, using an automated BP device (OMRON, Kyoto, Japan). Following an over-night fast (12-h), venous blood samples were collected for the measurement of plasma concentrations of free fatty acids (FFA, mmol/l), total-cholesterol (TC, mmol/l), HDL-cholesterol, mmol/l, LDL-cholesterol, mmol/l, triglycerides, mmol/l, glucose, mmol/l, and insulin, mU/l. Fasting plasma glucose concentration were analysed using the glucose oxidase method (YSI 2300 STAT PLUS; YSI Life Sciences, Yellow Springs, OH, USA), and blood lipids were measured using the Roche modular autoanalyzer and enzymatic colorimetric assays were used to analyze total cholesterol, triglycerides, and HDL-C concentrations. The LDL-C concentrations were determined using the Friedewald formula [26]. Insulin levels were determined by a Micro particle Enzyme Immunoassay (MEIA) (AxSym Insulin Kit, Abbott, IL, USA).

### Insulin sensitivity

The homeostasis model assessment (HOMA) of insulin resistance was calculated from fasting glucose and insulin levels [27]. A cut-off point of 1.95 for HOMA-IR was used to group subjects according to risk (≤ 1.95 for insulin sensitive (IS), > 1.95 for insulin resistant (IR) groups [28].

### Habitual energy and nutrient intake

All participants completed a previously validated, structured food frequency [29] questionnaire (Dietary Assessment and Education Kit, Medical Research Council of South Africa, South Africa) administered by a registered dietician. Energy intake (EI) was analysed using the computer package FoodFinder™3 software application (Version 1, Medical Research Council of South Africa). Total energy intake (TEI) (kJ), total carbohydrate (CHO), protein, fat and alcohol (g and % of energy) were calculated. The food quotient (FQ) was calculated from the food records, with the macronutrient composition expressed in percentage, using the following equations:

(1) $$\begin{array}{l} \text{FQ}=\left(0.207\times \mathrm{carbohydrate}\left(\%\right)+0.159\times \mathrm{fat}\left(\%\right)+0.193\times \mathrm{protein}\left(\%\right)+0.137\times \mathrm{alcohol}\left(\%\right)\right)\\ /\left(0.207\times \mathrm{carbohydrate}\left(\%\right)+0.226\times \mathrm{fat}\left(\%\right)+0.243\times \mathrm{protein}\left(\%\right)+0.206\times \mathrm{alcohol}\left(\%\right)\right) \end{array}$$

[30].

Excluding under/over reporters, only subjects with an EI: REE ≥ 1.05 and < 2.28, ie. adequate reporters, were included in this analysis [31]. Of the 140 included in the final sample, 69 were insulin sensitive, and 71 insulin resistant. The following reported dietary variables were also documented: carbohydrates, fats (poly & mono-unsaturated and saturated fats) and protein (animal and plant).

### Short fat questionnaire (SFQ)

The short fat questionnaire [32] is a brief, 17-item self-administered questionnaire which provides a measure of habitual dietary fat consumption. Fifteen of the questions are worth 0 to 4 points, whereas the remaining two can yield a maximum of 2 points each. Finally, the scores of all items are summed to generate a total out of 64 which is interpreted as follows: 0–17 = low fat intake; 18–39 = moderate fat intake; ≥ 40 = high fat intake.

### Resting energy expenditure (REE), respiratory exchange ratio (RER) and metabolic flexibility measured by RER:FQ ratio

Resting energy expenditure (REE) was measured using indirect calorimetry (Sensor Medics VMAX ventilated hood indirect calorimeter, Yorba Linda, CA) following a 12-h overnight fast. Subjects were instructed to report to the laboratory very early in the morning and rested in the supine position for 20-min. Subjects were tested in a darkened, thermo-neutral room and instructed to refrain from any movement for the duration of the assessment. The measurement period was 30 min, to allow time to reach and maintain a metabolic steady state.

Respiratory exchange rate (RER) and energy expenditure (EE) were averaged from the last 10 min of each assessment. Energy expenditure was converted into kilojoules using the Weir equation [33], and expressed on a per minute basis. The estimated fat and carbohydrate oxidation rates were calculated using the average respiratory exchange ratio (RER). The proxy measure for metabolic flexibility in the present study was the fasting RER:FQ ratio. A value of greater than 1, suggested that fat oxidation was lower relative to the fat intake in the diet.

### Demographic, medical history, socio-cultural and behavioural factors

Subjects completed a demographic questionnaire, determining socio-economic status on the basis of factors such as: education, housing and housing density and occupation. Physical activity was self-reported and assessed using the previously validated Global Physical Activity Questionnaire (GPAQ) [34].

In addition, subjects reported personal and family medical history and were subsequently grouped according to family history of obesity (FH OB) and family history of diabetes mellitus (FH DM), on the basis of one or more of their immediate family members having the condition.

### Statistics

The data were analyzed using Statistica Version 9 (StatsSoft Inc. Version 8, Tulsa, OK, USA) and expressed as means and standard deviations. Data that were not normally distributed were log-transformed where necessary. The insulin sensitive women were significantly older than the insulin resistant women. Therefore, all analyses were subsequently age-adjusted. Insulin sensitive and insulin resistant groups were compared, with respect to anthropometry, dietary intake and reported energy expenditure and metabolic outcomes, using analyses of covariance. Combining the two groups (Insulin sensitive and Insulin resistant), pearson-product moment correlations were used to identify variables that were associated with RER and HOMA-IR. Multiple regression analysis was used to determine factors that were independently associated with insulin resistance in our study sample including age, metabolic flexibility, body composition, diet and physical activity. HOMA-IR was log-transformed for the analysis.

Analyses of covariance were subsequently used to compare insulin-resistant and insulin-sensitive groups, with respect to RER, dietary intake, and physical activity as well as comparing those persons with and without family history diabetes mellitus (FH DM), adjusting for age and body composition. An alpha level of less than or equal to 0.05 was accepted as statistically significant.

## Results

### Subject characteristics

The subject characteristics of the study population are presented in Table 1. The insulin sensitive group was younger and more physically active than the insulin resistant group (p < 0.001). The insulin resistant group had a higher body fat %, waist circumference (cm), VAT (cm^2^) and SAT (cm^2^). However, the insulin sensitive group had a higher VAT/SAT ratio (p < 0.001).

**Table 1:** Subject characteristics and body composition

	**IS**	**N**	**IR**	**N**	**P value**
**Age (years)**	31±7.6	69	31±8.6	71	<0.001
**Weight (kg)**	61±6.4	69	91.6±15	71	<0.0001
**BMI(kg/m**^**2**^**)**	21.5±1.8	69	33±4.8	71	<0.0001
**Waist (cm)**	76.8±6.4	69	103±11.1	71	<0.0001
**Body fat (%)**	27.9±5.02	66	43±5.3	70	<0.0001
**VAT (cm**^**2**^**)**	61.2±20.1	55	141.9±61	58	<0.0001
**SAT (cm**^**2**^**)**	165±62.5	55	487±142.5	58	< 0.0001
**VAT/SAT**	0.41±0.2	55	0.30±0.11	58	<0.001
**MET**^**.**^**min/wk**	308±357	69	152±205	71	<0.05
**FH obesity (%)**	18(26%)	69	26(36.6%)	71	0.07
**FH DM (%)**	10(14.4%)	69	19(26.76%)	71	<0.05

Results from analysis of covariance, ANCOVA, values are adjusted for age and expressed as means ± SD. BMI, body mass index; VAT, visceral adipose tissue; SAT, subcutaneous adipose tissue, FH, Family History; DM, Diabetes Mellitus.

### Metabolic risk parameters

Fasting plasma [glucose], and serum [insulin], and HOMA-IR, presented in Table 2, were significantly higher in the IR group, by design. [Total cholesterol], [LDL-chol], [triglycerides], and TC/HDL ratio were also significantly higher in the IR group compared to the IS group (even after adjusting for age).

**Table 2:** Markers of metabolic risk between IS and IR groups

	**IS**	**N**	**IR**	**N**	**P value**
**Fasting [glucose] (mmol/l)**	4.5 ± 0.3	69	4.8 ± 0.4	71	<0.0001
**[Insulin] (mU/l)**	4.8 ± 1.3	69	12.7 ± 4.7	71	0.01
**[FFA] (mmol/l)**	0.33 ± 0.2	69	0.31 ± 0.2	70	NS
**HOMA-IR**	0.98 ± 0.3	69	2.71 ± 1.1	71	<0.0001
**Total [cholesterol] (mmol/l)**	4.6 ± 0.8	69	4.9 ± 1.0	71	0.033
**[LDL-C] (mmol/l)**	2.5 ± 0.8	69	3.0 ± 0.85	71	<0.0001
**[HDL-C] (mmol/l)**	1.8 ± 0.4	69	1.5 ± 0.4	71	<0.0001
**[Triglycerides] (mmol/l)**	0.90 ±0.4	69	1.17 ± 0.6	71	<0.0001
**TC/HDL**	2.6 ± 0.7	69	3.5 ± 1.1	71	<0.0001
**TG/HDL**	0.5 ± 0.26	67	0.86 ± 0.6	71	<0.0001

Results from ANCOVA, values are adjusted for age and expressed as the means ± SD.

FFA, free fatty acids; HOMA-IR,homeostasis model for insulin resistance; LDL-C.

low-density lipoprotein-cholesterol,HDL-C,high-density lipoprotein-cholesterol.

TG, triglycerides.

### Habitual dietary intake

The habitual reported dietary intake of both groups is presented in Table 3. Total reported energy intake (MJ/day) (p < 0.05), and dietary fat (g) intake were significantly higher in the insulin resistant group, compared to the insulin sensitive group. Further, saturated fat (% of total energy), mono-and poly-unsaturated fat % (% of total energy) and ω-6 fatty acids (g) were also significantly higher in the insulin resistant group (p < 0.05, data not shown). In contrast, there was no significant difference in the food quotient between groups.

**Table 3:** Habitual dietary Intake, energy expenditure and substrate oxidation

	**IS**	**IR**	**P value**
	**(N=69)**	**(N=71)**	
**Energy and macronutrient intake**			
**Energy Intake (MJ/d)**	9.1±1.9	9.7±1.9	<0.05
**Carbohydrate (g)**	238.2±62.7	263.1±66.4	0.02
**Protein (g)**	80.2±19.4	131.2±17.9	0.04
**Fat Intake (g)**	75.2±23.4	79.6 ±24.2	<0.05
**Food Quotient (FQ)**	0.86±0.02	0.86±0.02	NS
**Energy expenditure and substrate oxidation**			
**RER**	0.87±0.1	0.89±0.1	NS
**RER/FQ**	1.01±0.1	1.03±0.1	NS

Results of ANCOVA, values are expressed as the means ± SD; MJ/day, mega joules per day; RER, respiratory exchange ratio; FQ, food quotient.

### Resting energy expenditure and substrate oxidation

Unadjusted resting energy expenditure values were significantly lower in the insulin resistant group (p < 0.001, Table 3). However following adjustment for free fat mass, resting energy expenditure between groups was not different. Similarly we found no differences in the RER and RER: FQ ratio between groups.

### Factors associated with substrate oxidation (RER), insulin resistance (HOMA-IR) and metabolic flexibility (RER:FQ)

When combining the groups (Table 4), we showed that certain lifestyle variables (lower fat oxidation higher respiratory exchange ratio, lower RER:FQ ratio) and body composition (higher body fat %, and subcutaneous adipose tissue, higher visceral adipose tissue and waist circumference) were associated with HOMA-IR. We also found significant inverse associations between specific ω-3 dietary fatty acids, eicosapentaenoic acid (C20:5; EPA, r=−0.22, P < 0.009), docosapentaenoic acid (C22:5, DPA, r=−0.27, P < 0.001), docosahexaenoic acid (C22:6; DHA, r=−0.29, P < 0.0001), and resting energy expenditure. A higher habitual intake of these dietary fatty acids was associated with an overall higher rate of fasting fat oxidation. In addition, RER:FQ ratio was significantly correlated to other markers of metabolic risk including [HDL-C] (r = − 0.19, P = 0.03), and [TG/HDL] ratio, (r = 0.19, P = 0.03).

**Table 4:** Dietary and lifestyle factors associated with HOMA-IR

	**N**	**r**	**P value**
**Added Sugar (g)**	140	0.20	0.005
**% Total Energy(Sugar)**	140	0.20	0.008
**Insoluble fibre (g)**	140	−0.25	0.003
**Soluble fibre (g)**	140	−0.26	0.002
**ω-6 FA (g)**	140	0.21	0.01
**% Energy Plant Protein**	140	−0.23	0.006
**Physical activity (MET**^**.**^**min/week)**	140	−0.29	0.001
**Short Fat Questionnaire (pts)**	140	0.28	0.001
**RER**	140	0.21	0.01
**RER/FQ**	140	−0.19	0.03
**Body Fat %**	136	0.56	0.0001
**Total SAT (cm**^**2**^**)**	113	0.60	0.0001
**VAT (cm**^**2**^**)**	113	0.54	0.0001
**VAT/SAT**	113	−0.20	0.02
**Waist (cm)**	140	0.60	0.0001

Results of Pearson-product moment correlations.

We subsequently performed a multiple regression analysis to determine factors that were independently associated with insulin resistance (Table 5). Age, free fatty acids, METS, visceral adipose tissue and total body fat (%), family history of diabetes, as well as RER:FQ, accounted for 50% of variance in HOMA-IR (log transformed) (p < 0.00001).

**Table 5:** Forward, stepwise, multiple linear regression for log HOMA-IR

	**Partial correlation coefficients**	**Β Parameter estimates**	**Std Error**	**P value**
**Intercept**		−1.06	0.29	0.0005
**Age (years)**	−0.31	−0.010	0.003	0.0002
**%Body Fat**	0.41	0.012	0.003	0.0001
**VAT**	0.32	0.00137	0.0005	0.005
**RER/FQ**	0.23	0.839	0.260	0.002
**FH DM**	0.16	0.0975	0.435	0.027
**[FFA]**	−0.15	−0.206	0.097	0.035
**Physical activity (MET**^**.**^**min/week)**	−0.08	−0.00008	0.00007	0.24

VAT, visceral adipose tissue; RQ, respiratory quotient; FH DM, family history of diabetes mellitus, FFA, free fatty acids, Adjusted R^2^ =0.50, SEE = 0.18, P < 0.00001.

**Figure 1:**
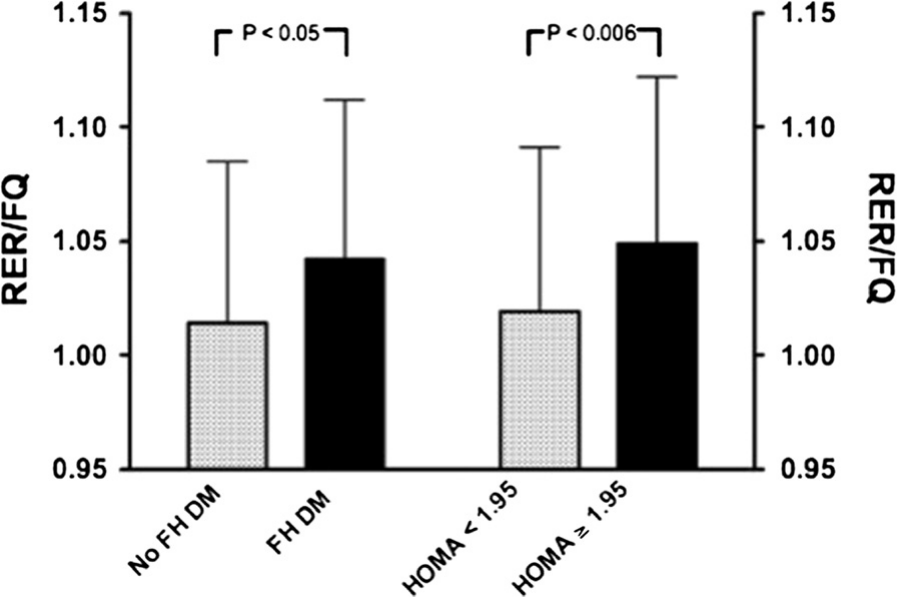
Fat oxidation in relation to dietary fat was lower (represented by a higher RER/FQ ratio) in persons with a family history of diabetes, and in those who were insulin resistant.

Furthermore, subjects who had a HOMA ≥ 1.95 had a higher RER:FQ ratio, as well as a higher resting energy expenditure than those with HOMA < 1.95 (p < 0.006), even after adjusting for differences in body fat % and age. Similarly, those with a family history of diabetes also had a higher RER:FQ ratio, after adjusting for differences in body fat percentage and age (p < 0.05) (Figure 1).

## Discussion

The results of the present study supported our hypothesis that metabolic inflexibility, indicated by a pragmatic proxy measure of fasting resting energy expenditure to food quotient (RER:FQ) ratio, was associated with insulin resistance and family history of diabetes mellitus, in otherwise apparently healthy women, independent of body fatness and age. Insulin resistance was associated with lower rates of whole body fat oxidation in relation to habitual dietary fat intake. This extends the findings of Ukropcova [19], who demonstrated impaired metabolic flexibility in response to short-term high-fat feeding, in healthy individuals, as measured by changes in resting energy expenditure during sleep or in response to a hyperinsulinaemic, euglycaemic clamp. Similar to the current study, this effect was most marked in those with a family history of Type 2 diabetes mellitus. However, unlike previous studies, the present study demonstrated these relationships using simple fasted resting measures of substrate oxidation, against self-reported, habitual dietary fat intake, without any manipulation of diet, or induced glycaemia [16].

The link between substrate oxidation or metabolic flexibility and family membership, as well as, family history of diabetes, has been demonstrated using 24-hr resting energy expenditure [14,30], fasting substrate oxidation [34] and suppression of fat oxidation in response to insulin-induced glycaemia and in response to high-fat feeding, Ukropcova [17,19], hypothesized that patterns of fat oxidation are genetically or epigenetically determined, in that, the differences in metabolic phenotype were independent of body fat percentage. Taken together, these results suggest that metabolic inflexibility is linked to genetic or epigenetic phenomenon, and is not secondary to insulin resistance, or the result of obesity, per se.

In previous studies, metabolic inflexibility has been shown to be concomitant with reduced skeletal muscle mitochondria size and density, [17,35] with an associated decrease in capacity for complete oxidative disposal of fat and loss of insulin sensitivity. Meex, [36] demonstrated that, 3 months of exercise training improved insulin sensitivity in both type 2 diabetics and non-diabetic individuals, along with metabolic flexibility, particularly in relation to insulin-stimulated, oxidative, glucose disposal. In addition, mitochondrial function was increased in both groups. This is consistent with the results of the current study, in which the total physical activity levels and RER:FQ ratio contributed to the overall variance in HOMA-IR, or insulin resistance (Table 5). However, we found no direct correlation between self-reported physical activity and resting energy expenditure or the RER:FQ ratio. The lack of a direct association between physical activity, fasting substrate oxidation and metabolic flexibility, as measured in the present study, may be related to the fact physical activity levels were generally low, and the data were not normally distributed. Alternatively subjects may have over reported physical activity levels or the GPAQ may not have been sufficiently sensitive to distinguish between groups. Not unexpectedly, we found that the insulin resistant group reported lower levels of physical activity and a higher dietary fat intake [37], and thus, may have additionally confounded this relationship in bivariate analyses.

One unexpected finding in the present study, was the inverse associations between serum free fatty acids concentrations and insulin resistance, in these non-diabetic, otherwise healthy women. While there are numerous studies that have shown that elevated free-fatty acid levels may be implicated in the aetiology of insulin resistance and are associated with Type 2 diabetes mellitus [37,38], there is also some indication that the sequelae of insulin resistance may be tissue- and substrate-specific [16,39]. As such, it is possible that in these non-diabetic subjects, the increased circulating insulin concentrations, were still effective in the suppression of lipolysis or in the non-oxidative disposal of fatty acids [40].

In a cohort study, Bickerton, [41] compared men in the highest and lowest quartiles for fasting plasma insulin and found that there were no differences between groups with respect to circulating free fatty acid concentrations. However, those with the highest fasting insulin also had higher triglyceride concentrations. In a smaller metabolic study, insulin-resistant men were compared to weight-matched, BMI-matched controls. Despite higher insulin levels, the insulin-resistant group had similar fasting free fatty acid concentrations when compared to control subjects, and systemic fatty acid production and appearance were lower. These authors suggest that there are ‘altered metabolic partitioning’ of fatty acids (reduced oxidation, increased re-esterification), in response to the insulin resistant state. This is consistent with Forbes [42], who found that lipolysis, palmitate appearance and palmitate oxidation were actually lower in non-obese women at risk for diabetes compared to controls. While at the same time, they demonstrated higher plasma insulin levels, suggesting increased capacity for storing fat.

The influence of diet on insulin sensitivity is mediated by both energy and nutrient content, in particular by different types of dietary fatty acids [42,43]. The higher dietary fat intake (Table 3) of the insulin resistant group compared to the insulin sensitive group directly relates to insulin sensitivity [44]. We also found that higher dietary ω-3 fatty acids, specifically C20:5, C22:5, and C22:6, were strongly associated with increased fat oxidation (data not shown). Thus, specific dietary fatty acids may modulate the relationship between substrate oxidation and insulin resistance, by altering fatty acid partitioning, towards either storage or oxidation [45].

One limitation of the present study was that total fat intake was assessed by a quantified validated food frequency questionnaire (FFQ) [29], rather than an objective biomarker, which may have introduced the potential for bias, as over and under reporters were excluded from the analysis. However, a study in a sample of American men comparing fatty acid intake assessed by subcutaneous fat aspirate [46], 2-weeks diet records, and an amended version of the food frequency questionnaire used in the US Nurses’ Health Study [47] suggested that the estimates of fat intake from the food frequency questionnaire were as valid as those from the diet records.

## Conclusion

We found that a pragmatic measure of metabolic flexibility based on fasting respiratory exchange ratio in relation to reported habitual dietary fat intake was a useful marker of insulin resistance in otherwise, healthy women, independent of obesity. Importantly, metabolic flexibility, measured in this way, was lower in those women who reported a family history of diabetes mellitus. Further research is needed to characterize the oxidative response to various dynamic challenges to better clarify the role of metabolic flexibility in the patho-physiology of obesity and diabetes. Additionally, longitudinal studies, monitoring changes in substrate oxidation in relation to substrate balance, over time, and in relation to changes in body energy stores and fat distribution are needed. Finally, there is a need for a greater understanding of the respective role of genotype and environment in determining this susceptible phenotype.

## Competing interests

The author declares that she has no competing interests to declare.

## Authors’ contributions

MC helped to design the study, collected the data, analysed and interpreted the results, wrote and edited the manuscript. JE was involved in subject recruitment, data collection and analysis and interpretation of the results, and editing the manuscript. JG and EVL were involved in the design of the study, analysis and interpretation of the data, writing and editing of the manuscript. LD, JK and NL were involved in the analysis and interpretation of the results, and the editing of the manuscript. All authors read and approved the final manuscript.
